# Supplementary material for: Efficacy of the Aqueous Extract of Azadirachta indica Against the Marine Parasitic Leech and Its Phytochemical Profiling
Source: Molecules. 2021 Mar 29;26(7):1908. doi: 10.3390/molecules26071908 (PMC8037938; doi:10.3390/molecules26071908)
Supplement: Supplementary file 1 [file molecules-26-01908-s001.pdf]

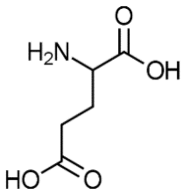

[1]

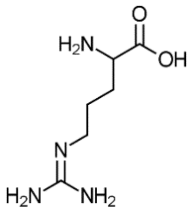

[2]

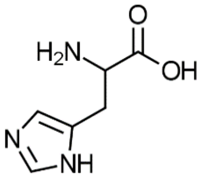

[3]

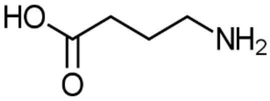

[4]

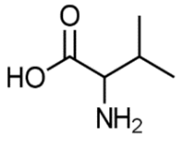

[5]

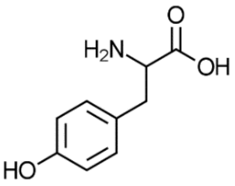

[6]

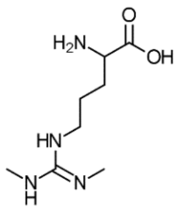

[7]

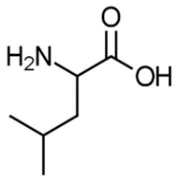

[8]

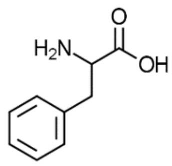

[9]

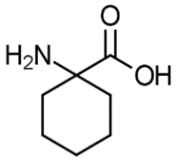

[10]

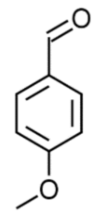

[11]

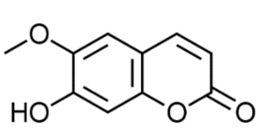

[12]

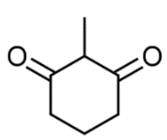

[13]

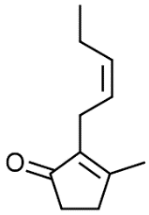

[14]

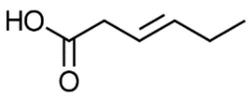

[15]

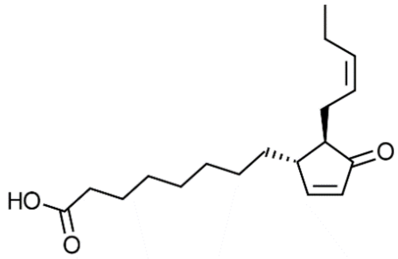

[16]

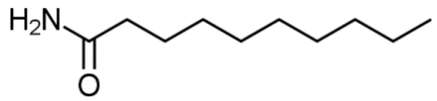

[17]

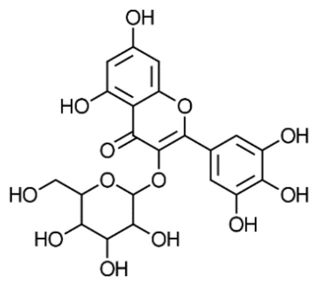

[18]

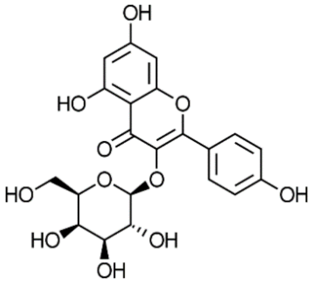

[19]

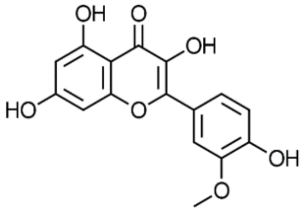

[20]

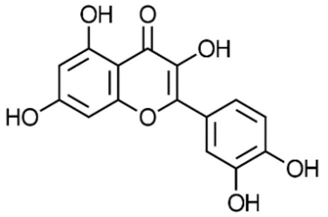

[21]

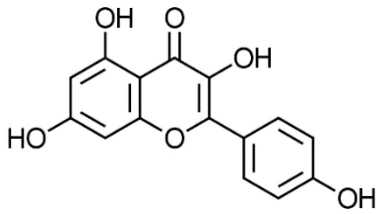

[22]

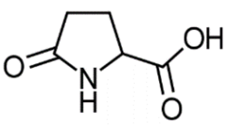

[23]

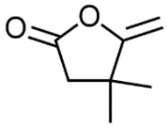

[24]

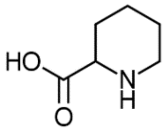

[25]

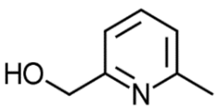

[26]

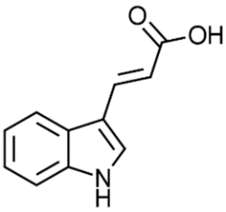

[27]

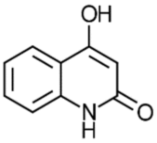

[28]

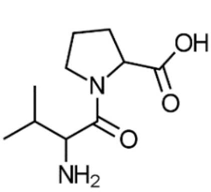

[29]

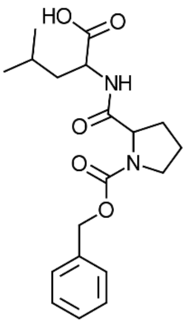

[30]

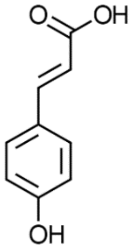

[31]

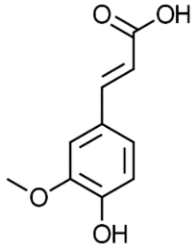

[32]

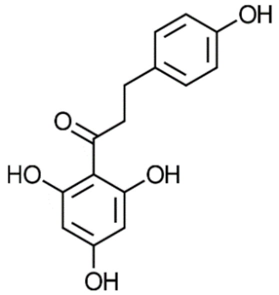

[33]

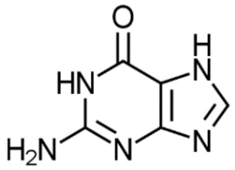

[34]

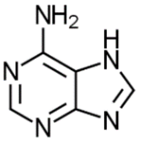

[35]

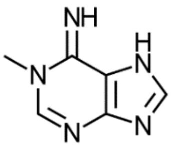

[36]

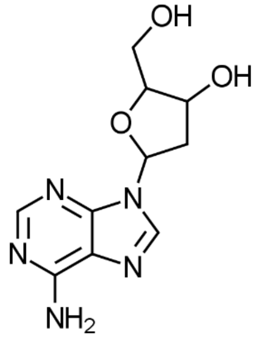

[37]

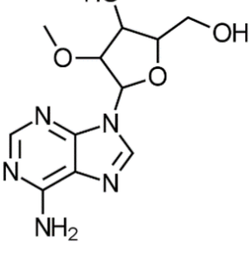

[38]

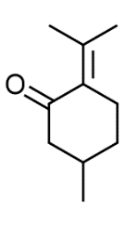

[39]

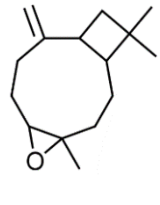

[40]

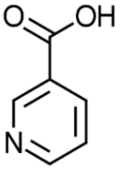

[41]

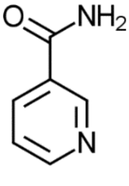

[42]

Figure S1. Structures of the identified compounds **(1–42)** from the extract of *Azadirachta indica,* compound names are given in Table 3.

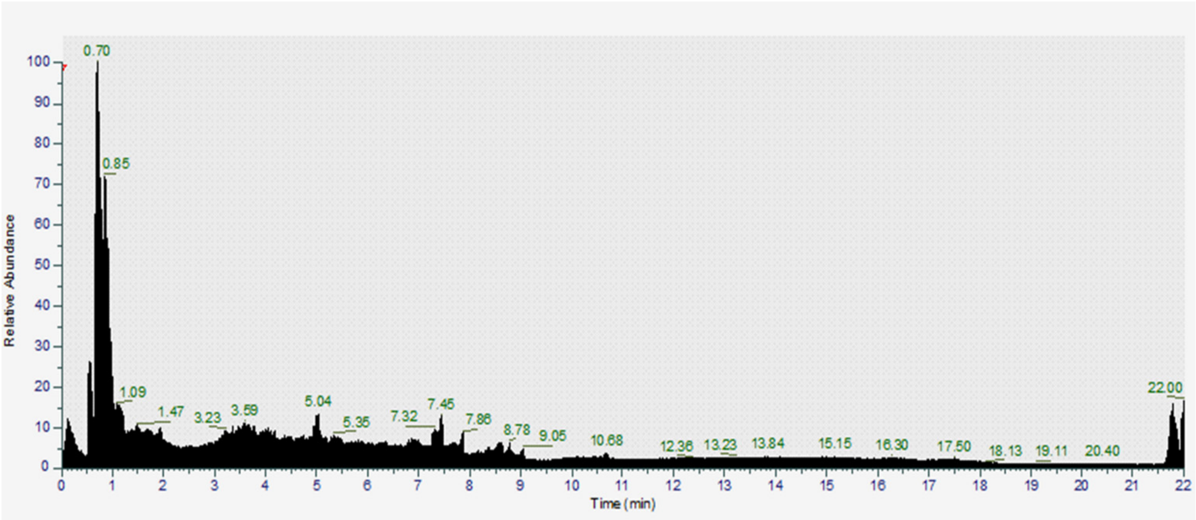

Figure S2. Base peak chromatogram (BPC) of the aqueous extract of *Azadirachta indica*
